# Plasma homocysteine in adolescents depends on the interaction between methylenetetrahydrofolate reductase genotype, lipids and folate: a seroepidemiological study

**DOI:** 10.1186/1743-7075-6-39

**Published:** 2009-10-05

**Authors:** Ruth Gil-Prieto, Valentín Hernández, Beatriz Cano, Manuel Oya, Ángel Gil

**Affiliations:** 1 Preventive Medicine & Public Health Unit, Health Sciences I Department, Rey Juan Carlos University Avda de Atenas s/n, 28922 Alcorcón, Madrid, Spain; 2 Lipids Unit, Jiménez Díaz Fundation, Madrid, Avda Reyes Católicos, 2, 28040 Madrid, Spain

## Abstract

**Background:**

Many publications link high homocysteine levels to cardiovascular disease. In Spain there is little information on the prevalence of hyperhomocysteinaemia and associated vitamin factors among the general population, and less still among children. Cardiovascular risk factors in the childhood population may be related to the appearance of cardiovascular disease at adult age. The aim of this study is to establish a definition of hyperhomocysteinaemia in adolescents and to analyze the influence of vitamin and metabolic factors in homocysteine levels in this population group.

**Methods:**

Descriptive, cross-sectional epidemiological study to estimate serum homocysteine, vitamin B12 and folate levels, as well as plasma total, HDL- and LDL- cholesterol in a schoolgoing population aged 13 to 17 years in Madrid, Spain.

Spearman correlation analysis was performed to ascertain quantitative comparison, Pearson's χ2 test (frequency < 5, Fisher) was used for comparison of prevalences, Mann-Whitney U and Kruskal-Wallis test were used for comparison of means and Bonferroni correction was used for post-hoc tests. A multivariate logistic regression model was performed in the multivariate analysis.

**Results:**

Based on the classic values for definition of hyperhomocysteinaemia in adults, prevalence of hyperhomocysteinaemia in the study population was: 1.26% for 15 μmol/L; and 2.52% for 12 μmol/L.

Deficits in HDL cholesterol and serum folate levels yielded adjusted Odds Ratios (OR) for hyperhomocysteinemia of 2.786, 95% CI (1.089-7.126), and 5.140, 95% CI (2.347-11.256) respectively. Mutation of the methylenetetrahydrofolate reductase (MTHFR) C677T genotype also raises the risk of hyperhomocysteinaemia (CC→CT: OR = 2.362; 95% CI (1.107-5.042) CC→TT: OR = 6.124, 95% CI (2.301-16.303))

**Conclusion:**

A good definition of hyperhomocysteinaemia in adolescents is the 90^th ^percentile, equivalent to 8.23 μmol/L. Risk factors for hyperhomocysteinaemia are cHDL and folate deficiency, and the MTHFR C677T mutant genotype. No significant effect could be assessed for vitamin B_12_. Coexistence of all three factors increases the risk of suffering from hyperhomocysteinaemia 87-fold.

## Background

In addition to being the principal cause of premature death in most European countries, cardiovascular diseases (CVDs) are a major cause of disability, and, by extension, increased health care cost [1]. Such diseases are generally due to a combination of several risk factors [2]. In 1996, the 27^th ^Bethesda Conference proposed hyperhomocysteinaemia as one of the new cardiovascular risk factors [3,4].

Inasmuch as it involves a potentially modifiable and correctable risk factor, this hypothesis is of great health care importance since it opens the door to a new form of intervention against cardiovascular diseases, such as dietary supplementation with group B vitamins and folic acid.

Homocysteine is a sulphur-containing amino acid derived from methionine, present in proteins of animal origin ingested as part of dietary intake. It is produced by intracellular demethylation of methionine and then exported to the plasma, where it circulates, mainly in its oxidised form.

Homocysteine is metabolised to methionine via remethylation or to cysteine via transulphuration at the hepatic level. During vitamin B_6_-dependent transulphuration, homocysteine is irreversibly catabolised into cysteine, thanks to the action of cystathionine-β-synthetase enzyme, in the presence of serine. Most of the homocysteine is remethylated, regenerating methionine, mainly due to the action of methionine synthetase, the enzyme that depends on the action of methylcobalamine (vitamin B_12_) as a co-factor and folate, in the form of 5-methyl-tetrahydrofolate, as a methyl group donor.

The enzyme, 5,10-methyl-tetrahydrofolate reductase (MTHFR) reduces 5,10-methylenetetrahydrofolate to 5-methyl-tetrahydrofolate in the presence of NADPH. This reaction is irreversible *in vivo *and its activity regulates the entry of folate into the homocysteine remethylation pathway.

The MTHFR C677T mutation is the most frequent cause of moderate hyperhomocysteinaemia due to genetic factors. The TT genotype has been found to have a direct effect on homocysteine concentration, with MTHFR enzymatic activity being reduced by as much as 50% in individuals with this mutation [5]. This polymorphism is present in its homozygous form in 5%-18% of the population [6].

The effect of the TT homozygote genotype on homocysteine concentration is not observed, however, when folic acid levels are elevated [7]. This is due to the fact that, despite the reduction in its enzymatic activity, MTHFR has sufficient 5-10 methyltetrahydrofolate substrate to generate the 5-methyltetrahydrofolate necessary for remethylation of homocysteine.

When these reactions are altered, homocysteine accumulates and is excreted into the blood, causing hyperhomocysteinaemia [8], which, according to a number of epidemiological studies, is associated with an increase in the probability of suffering from CVDs [9-15].

Hyperhomocysteinaemia is defined as elevated concentration of fasting plasma total homocysteine.

As is the case with other parameters, such as arterial hypertension, no threshold plasma homocysteine value has been established, above which the risk of suffering from a vascular disease is increased [16,17]. In most studies, this threshold is obtained arbitrarily, with values above the mean found in the control group plus two standard deviations [17-20], or above the 95th percentile, being used to define hyperhomocysteinaemia [16,17,20-22].

In Spain, Pijoan et al. constructed a reference interval (5-15 μmol/l) based on a sample of 396 apparently healthy individuals [23].

Plasma homocysteine levels increase with weight, height, body mass index, blood pressure, systolic and diastolic pressure, age, male gender and MHTFR 677TT genotype. In contrast, they decrease with high plasma folate and intraerythrocyte, vitamin B_6 _and vitamin B_12 _concentrations.

The established drug treatments for hyperhomocysteinaemia are vitamin supplements consisting of folate, vitamin B_6_, vitamin B_12 _or any combination of the three [24].

In the healthy paediatric population, homocysteine levels are influenced more by biochemical than by genetic factors, due to the high folate reserves present during childhood. No significant differences in homocysteine levels were observed between boys and girls until postpubertal age, when these rise in males [25].

Arterosclerosis begins at early ages of life [26,27] and environmental factors in childhood are known to be linked to the appearance of cardiovascular disease at adult age [28].

Determination of plasma homocysteine in children could have a predictive value vis-à-vis adult vascular risk [29].

## Methods

A descriptive, cross-sectional epidemiological study was conducted to estimate serum homocysteine levels in the schoolgoing population aged 13 to 17 years in the Madrid Region (*Comunidad de Madrid*). All children who participated (172 girls and 145 boys) were recruited at selected junior schools, using random, stratified, cluster sampling. Junior schools were selected in strata that would ensure representation of socio-economic differences.

For a level of precision of 3%, an estimated prevalence of 5% and a confidence level of 95%, a minimum sample size of 202 was needed. Assuming lost of 20%, a total of 243 subjects was needed. (EPI-INFO 2002; version 6).

The study included subjects of both sexes, who were aged over 13 years, attended schools in the Madrid Region and produced informed consent. The following were subjects excluded from the study: those whose parents failed to sign the authorisation to participate; those who were authorised but were nevertheless unwilling to participate; and those who presented with some type of acute or chronic illness that might affect the variables of interest.

In each case, data were collected on age, gender, physical activity, blood pressure and anthropometric variables.

### Anthropometric variables

Subjects' weight and height, as well as arm, waist and hip circumferences were measured in duplicate, using calibrated precision scales (Seca 812, precision 0.1 kg), portable wall-mounted stadiometres (Seca Ka We 4444 model) and flexible, inelastic, plastic belt-type measuring tapes fitted with a read-out buckle at one end. Body mass index (weight in kg/height^2 ^in m^2^) was then calculated on the basis of these measures.

### Blood pressure

Three readings were taken from each subject in accordance with standardised guidelines issued by international bodies, using a mercury sphygmomanometer (Diplomat Presameter Riester Model) and an appropriately sized cuff chosen in line with the individual's arm circumference.

### Lipid profile

Cholesterol and triglyceride levels were measured in total plasma through enzymatic methods.

In the case of girls, information was collected on the appearance or non-appearance of menarchy.

#### Processing of blood specimens

A first morning, 12-hour, fasting, 14-ml blood specimen was extracted from each subject by venipuncture, using a Vacutainer tube containing EDTA-Na2 as antioxidant and anticoagulant (10 ml EDTA) and an SST Vacutainer without anticoagulant (4 ml SST).

The specimens were then stored on ice and sent immediately to the laboratory for analysis, where they were centrifuged at 3500 rpm for 6 minutes a 4°C within the hour.

### Determination of total homocysteine

Total homocysteine in the fasting blood specimen [30] was determined, using an Abbott Diagnostics fluorescence polarisation immunoassay (FPIA). For multivariate analysis, higher decile (P90) was defined as hyperhomocysteinemia.

### Determination of vitamin B_12_

Vitamin B_12 _(Vit B_12_) in the fasting blood specimen [31] was determined, using a Roche Elecsys E170 MODULAR ANALYTICS immunoanalysis assay.

### Determination of folate

Folate in the fasting blood specimen [32] was determined, using a Roche Elecsys E170 MODULAR ANALYTICS immunoanalysis assay.

### Determination of the MTHFR C677T genotype

Replacement of cytosine by thimine at nucleotide position 677 converts the amino acid, alanine, into valine: (normal genotype = CC, heterozygous genotype = CT, mutated homozygous genotype = TT). DNA was amplified by PCR in a PTC-100 thermal cycler using nucleotides:

Forward: 5'TGAAGGAGAAGGTGTCTGCGGGA 3'

Reverse: 3'AGGACGGTGCGGTGAGGAGGTG 3'.

The product of this reaction was digested with HinfI restriction enzyme, and the fragments then separated in non-denaturing polyacrylamide gel.

For a negative control, distilled water instead of DNA in the reaction system was used for each panel of PCR. For positive DNA control, DyNAzyme DNA Polymerase Kit (Finnzymes Oy) was used.

#### Statistical data analysis

Based on all the valid data obtained, we performed a descriptive analysis of both the independent and dependent variables of interest, using centralisation and dispersion measures (means or medians where the distribution was asymmetric), accompanied by their corresponding 95% confidence intervals in the case of quantitative variables and distribution of frequencies (prevalences and proportions with 95% confidence intervals) in the case of qualitative variables.

This analysis was stratified by gender, presence of menstruation, age group, and MTHFR C677T genotype.

The normality of the homocedasticity variables was verified using the Kolmogorov-Smirnov test. Where variables did not follow a normal distribution, non-parametric tests were used. Homoscedasticity or homogeneity of variances was examined using Levene's test.

A Spearman correlation analysis was performed to ascertain the magnitude of the linear relationship between the quantitative anthropometric, biochemical and metabolic cycle variables and homocysteine values. The technique used for comparison of prevalences was Pearson's χ2 test (frequency < 5, Fisher). We used the Mann-Whitney U test for comparison of means, and the Kruskal-Wallis test for variables with more than two categories or for inclusion of different variables simultaneously. Bonferroni correction was used for *post-hoc *tests to ensure that all comparisons were made with α = 0.05.

Independent variables that displayed a significant association in the simple analysis as well as those that were clinically linked to the study variable, were included in a multivariate logistic regression model. Based on this, the adjusted Odds Ratios of the principal study factors were obtained, along with their corresponding 95% confidence intervals.

In all hypothesis testing to determine differences, associations and relationships were deemed significant at *p *< 0.05.

Statistical data analyses were performed using the SPSS computer software programme (Statistical Package for Social Sciences) version 16.0.

#### Ethical aspects

The research protocol was approved by the Jiménez Díaz Foundation Clinical Research Ethics Committee. This study complied in all respects with the ethical safeguards laid down by the Helsinki Declaration and its subsequent updates, and with Spanish legislation governing clinical research involving humans and protection of data of a personal nature

## Results

The study population totalled 317 students, 50% aged 13 years, 35% aged 14 years and 15% aged 15 or more years; 54% were females and 46% males. The mean age of the study subjects was 13.69 years, 95% CI (13.60-13.78), and their anthropometric characteristics corresponded to the expected values for their age.

Table 1 shows the serum or plasma values for the principal biochemical variables and possible hormonal factors implicated in cardiovascular disease. Mean concentrations were as follows: serum folate, 7.83 nM, 95% CI (7.42-8.23) nM; vitamin B_12_, 503 pM, 95% CI (478-528) pM; and homocysteine, 4.92 μM, 95% CI (4.50-5.35) μM.

**Table 1:** Biochemical variables and hormonal factors

	**Mean**	**95% Confidence Interval**
Glucose (mg/dl)	90.9	90.0-91.9
Total cholesterol (mg/dl)	160.9	157.9-163.9
HDL cholesterol (mg/dl)	47.3	46.0-48.7
LDL cholesterol(mg/dl)	96.7	94.0-99.4
Total cholesterol/cHDL	3.60	3.48-3.71
cLDL/cHDL	2.20	2.10-2.30
Menarche age (years)	12.01	11.86-12.15

Folate (nmol/L)	7.83	7.42-8.23
Vitamin B_12 _(pmol/L)	503	478-528
Homocysteine (μmol/L)	4.92	4.50-5.35

HDL: High Density Lipoproteins

LDL: Low Density Lipoproteins

Taking the classic values as reference, prevalence of hyperhomocysteinaemia in the study population was 1.26% for 15 μmol/L, and 2.52% for 12 μmol/L. Table 2 defines different cut-off points for hyperhomocysteinaemia in children.

**Table 2:** Threshold determination to define hyperhomocysteinemia

**Hyperhomocysteinemia**	**P50**	**P75**	**P90**	**P95**	**Hcy ≥ 12**	**Hcy ≥ 15**
**Threshold [Hcy]/μM**	4.30	5.93	8.23	10.41	12.00	15.00
**Number of cases**	157	78	32	15	8	4
**Prevalence %**	49.53	24.61	10.1	4.73	2.52	1.26
**95% CI**	43.99-55.06	19.84-29.37	6.78-13.41	2.38-7.08	0.79-4.26	0.03-2.50

P: percentile

When analysing MTHFR C677T genotype, normal homozygous CC in the methylenetetrahydrofolate reductase enzyme was present in 37.8%, heterozygous CT genotype was present in 47.3%, and TT genotype, corresponding to the homozygous mutation, was present in 14.9% of cases.

Table 3 shows the prevalence of potential cardiovascular risk factors and variables implicated in homocysteine metabolism. Of the total study population, 23.8% displayed deficient serum folate levels (≤ 5.3 nmol/l) and 24.6% displayed deficient vitamin B_12 _levels (≤ 336 pmol/l). Overweight, elevated glucose levels, deficient vitamin B_12 _and HDL cholesterol values were greater in boys, whilst girls showed greater sedentariness and hypercholesterolaemia. Prevalence of occasional tobacco and alcohol use increased with age. Folate deficit was significantly greater among subjects that displayed total (homozygote TT) mutation of the MTHFR C677T genotype (Table 3).

**Table 3:** Prevalence of cardiovascular risk factors and lipid profile

	**Total (%)**	Gender*		Age*			MTHFR C677T genotype*		
		
		**Male**	**Female**	**13**	**14**	**>14**	**CC**	**CT**	**TT**
Overweight	30.3	35.9	25.6	ns			ns		
Hypertension	8.5	ns		ns			ns		
Glucose (≥ 100 mg/dl)	15.3	22	9.6	ns			ns		
Total cholesterol (≥ 200 mg/dl)	7.0	2.8	10.5	ns			ns		
HDL cholesterol (≤ 35 mg/dl)	13.8	20.1	8.3	ns			ns		
LDL cholesterol (≥ 130 mg/dl)	6.4	ns		ns			ns		
Total cholesterol/cHDL (>5en ♂/4.5 en ♀)	9.1	ns		ns			ns		
cLDL/cHDL (>3.5 ♂/3 ♀)	8.5	ns		ns			ns		
Smoking	7.6	ns		3.2	14.5	19.1	ns		
Alcohol	9.8	ns		1.9	12.5	12.8	ns		
Sedentariness	17.7	9	25.1	ns			ns		
Folate (≤ 5.3 nmol/l)	23.8	ns		ns			18.8	20.4	46.7**
Vitamin B_12_ (≤ 336 pmol/l)	24.6	30.3	10	ns			ns		

*Statistically significant p < 0.05. ns: non-significant; ** differences in folate deficit were statistically significant (p < 0.001) only for homozygotic TT mutation. CT heterozygotic mutation does not show an statistically significant difference (p = 0.758)

HDL: High Density Lipoproteins

LDL: Low Density Lipoproteins

MTHFR: methylenetetrahydrofolate reductase

C: Cytosine; T:Thymine

In order to assess analyze the influence of vitamin and metabolic factors in homocysteine levels in this population group, hyperhomocysteinemia was defined as percentile 90^th^. Crude analysis showed how prevalence of hyperhomocysteinaemia increases with male gender (32.4% vs. 18.0% in female), age (21.0%, 22.3%, 40.4% for 13, 14 and >14, respectively), mutation of the MTHFR C677T genotype (15.1%, 24.8%, 46.8% for CC, CT and TT genotype, respectively), HDL cholesterol (22,7% vs. 37.2% for normal and low levels, respectively) and deficient folate values (19.4 vs. 43.2% for normal and low levels, respectively). These independent variables, as well of other of interest as vitamin B_12_, were included in a multivariate logistic regression model, obtaining adjusted Odds Ratios for the principal study factors (Table 4).

**Table 4:** Adjusted ORs for hyperhomocysteinemia

		**Odds Ratio**	**95% CI**	**p**
cHDL deficit*,**		2.786	1.089-7.126	0.033
Folate deficit*,***		5.140	2.347-11.256	<0.001
MTHFR C677T genotype *				
	CT	2.362	1.107-5.042	0.001
	TT	6.124	2.301-16.303	

*Statistically significant.

HDL: High Density Lipoproteins

MTHFR: methylenetetrahydrofolate reductase

C: Cytosine; T:Thymine

**<5.3 nmol/l

***<35 mg/dl

Deficient HDL cholesterol and serum folate levels registered adjusted Odds Ratios of OR = 2.786, 95% CI (1.089-7.126) and OR = 5.140, 95% CI (2.347-11.256) respectively. The MTHFR C677T mutant genotype raised the risk of hyperhomocysteinaemia (CC→CT: OR = 2.362; 95% CI (1.107-5.042) CC→TT: OR = 6.124, 95% CI (2.301-16.303))

After plausible biologically interactions have been ruled out, the multiplicative nature of the association among several risk factors in the sample can be observed, namely, prevalence of hyperhomocysteinaemia increases in accordance with the number of risk factors (FR) that accumulate.

**Figure 1:**
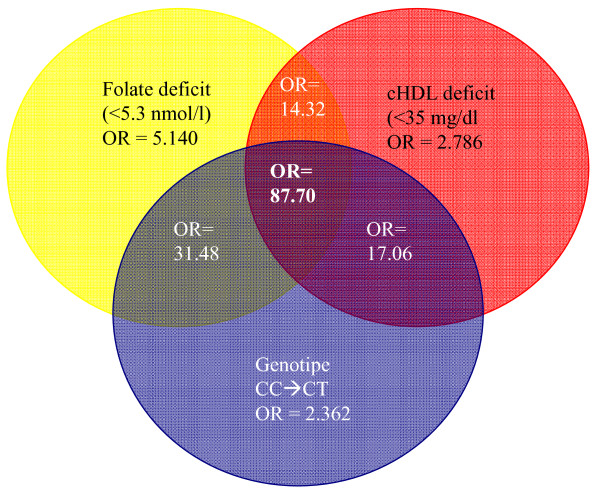
**Cumulative ORs: Folate, cHDL and MTHFR C677T genotype**. Cumulative effect of risk factors in homocystein prevalence. HDL: High Density Lipoproteins. MTHFR: methylenetetrahydrofolate reductase. C: Cytosine; T: Thymine; CC: normal genotype; CT: partial mutation; TT: mutation in homozygosis.

From Table 5, it will be seen how the prevalence of hyperhomocysteinaemia rises when deficient folate and HDL cholesterol values and mutant genotype coincide. Shown in Figure 1 is the cumulative effect of the most important risk factors, i.e., genotype, folate deficit and HDL cholesterol deficit. An indiviual with CT genotype and deficient folate and cHDL is almost 34 times more likely to suffer from hyperhomocysteinaemia; and if that same individual has these deficits plus the TT genotype, the likelihood of his/her having high serum homocysteine levels is then 87 times higher.

**Table 5:** Risk factor accumulation: genotype, folate and HDL-cholesterol

**Genotype**	**Folate**	**cHDL**	**% Hyperhomocysteinemia**
CC	Normal	Normal	10.7
		
		Low levels**	18.2
	
	Low levels*	Normal	31.8
		
		Low levels**	0.0

CT	Normal	Normal	20.2
		
		Low levels**	36.8
	
	Low levels*	Normal	29.2
		
		Low levels**	66.7

TT	Normal	Normal	38.1
		
		Low levels**	33.3
	
	Low levels*	Normal	55.6
		
		Low levels**	100.0

HDL: High Density Lipoproteins

MTHFR: methylenetetrahydrofolate reductase

C: Cytosine; T:Thymine

*<5.3 nmol/l

**<35 mg/dl

## Discussion

This study suffers from the limitations inherent in all cross-sectional studies, in that it does not allow for causal or temporal relationships among variables to be established, thus rendering it necessary for subsequent analytical epidemiological studies to be conducted in order to confirm the results and hypotheses generated. Furthermore, some confounding variables may not have not been included in this study, e.g., methionine intake, creatinine levels, or polymorphisms of the nicotinamide-methyltransferase enzyme (chromosome 11q23), which have recently been linked to plasma homocysteine levels [33].

In Spain, Gutiérrez-Revilla et al. (n = 83), Vilaseca et al. (n = 195) and Mainou et al. (n = 80) have respectively published cross-sectional studies in children and adolescents. The first two targeted the population aged 0 to 18 years, and the third the population aged 5 to 18 years. Ours is the first study conducted in Spain and focused on the adolescent population to have a substantial sample size.

In Western countries, vitamin deficiencies are rather infrequent, but in populations with important vitamin deficiencies, plasma homocysteine levels rise until reaching age-adjusted hyperhomocysteinaemia prevalences of 73% in men and 41% in women, as shown by a recent study conducted in Iran [34].

The distribution of the various polymorphisms of the MTHFR C677T enzyme in our sample was very similar to that observed in other studies [35,36], i.e., Hiraoka, with 32.9%, 51.6% and 15.5%, in CC, CT and TT respectively [37], or Gutiérrez-Revilla et al., with 42.2%, 41% and 16.9%, in CC, CT and TT respectively [25]. The cardiovascular risk factors studied showed no significant variation according to MTHFR genotype, which only affects folate and serum homocysteine levels.

The mean homocysteine values obtained by us are very similar to those found in children and adolescents in Norway and the USA, namely, 5.25 and 5 μmol/l, respectively [38,39]. There is no international consensus regarding the establishment of a cut-off point for defining hyperhomocysteinaemia, and there are still fewer data relating to the adolescent population. Prevalence of hyperhomocysteinaemia in the study population was 1.26% for 15 μmol/L and 2.52 for 12 μmol/L, results very similar to those recently obtained in young adults aged 20-22 years in Tokyo (1.18% for > 15 μmol/L) [37]. Taking into account other international studies, which use percentiles for determining other magnitudes in childhood and adolescence, the 90^th ^percentile is considered a good cut-off point for defining hyperhomocysteinaemia in developed countries [29,40,41]. In our study, this cut-off allowed us to show a statistically significant increase of plasma homocysteine levels depending on the study variables. In line with other studies [5,23,42-46], prevalence of hyperhomocysteinaemia was seen to increase in our study with the MTHFR C677T mutant allele (2- and 6-fold for partial and total C→T mutation, respectively), serum folate deficit (5-fold) and cHDL deficit (2.7-fold). No significant association was found with vitamin B_12 _after adjusting by age, gender and CV risk factors.

It has been observed that the coexistence of several risk factors, none of which is necessarily elevated to an extraordinary degree, in any given individual can actually be more dangerous than the existence of a single factor, regardless of its magnitude [44,47]. In our study, as previously described in Guo et al [48], prevalence of hyperhomocysteinaemia increases when risk factors accumulate. The probability of being hyperhomocysteinemic is 5 times higher with deficit serum folate levels than it is with normal values. If, the person concerned also happens to be a carrier of the MTHFR TT polymorphism, this risk is then multiplied by 30 because his/her ability to metabolise homocysteine is very reduced; and if, in addition to this, such a person has HDL cholesterol levels below 35 mg/dl, the likelihood of his/her being hyperhomocysteinemic is multiplied by 90. These results suggest that the higher decile is an appropriate cut-off to define homocysteinemia in adolescents.

While many national and international studies link deficit serum folate levels to hyperhomocysteinaemia and suggest dietary supplementation as a preventive strategy for reducing cardiovascular risk [48-53], other authors contend that, as hyperhomocysteinaemia has not yet been shown to be an independent risk factor, dietary supplementation is not justified [54].

We thus feel that we are confronted by a still unresolved research question, and trust that our study may serve as a further contribution towards finding an answer in this field of investigation

## Conclusion

A good definition of hyperhomocysteinaemia in adolescents is the 90^th ^percentile, equivalent to 8.23 μmol/L.

Risk factors for hyperhomocysteinaemia are cHDL and folate deficiency, and the MTHFR C677T mutant genotype. Coexistence of all three factors increases the risk of suffering from hyperhomocysteinaemia 87-fold.

## Competing interests

The authors declare that they have no competing interests.

## Authors' contributions

RGP: design and coordination of the study, data acquisition, analysis and interpretation of data, perform the statistical analysis and drafted the manuscript, VH: participated in the statistical analysis, BC: carried out the PCRs, MO: design and coordination of the study, acquisition of funding, general supervision of the research group, AG: design and coordination of the study, acquisition of funding, general supervision of the research group. All authors but MO, which sadly died during manuscript preparation, read and approved the final manuscript.
